# Association between *APOE* Genotype with Body Composition and Cardiovascular Disease Risk Markers Is Modulated by BMI in Healthy Adults: Findings from the BODYCON Study

**DOI:** 10.3390/ijms23179766

**Published:** 2022-08-29

**Authors:** Ezgi Ozen, Rada G. Mihaylova, Natalie J. Lord, Julie A. Lovegrove, Kim G. Jackson

**Affiliations:** Hugh Sinclair Unit of Nutrition, Department of Food and Nutritional Sciences, and Institute for Cardiovascular and Metabolic Research and Institute for Food, Nutrition and Health, University of Reading, Whiteknights, Reading RG6 6DZ, UK

**Keywords:** *APOE*, BMI, body composition, DXA

## Abstract

Body mass index (BMI) has been suggested to play an important role in the relationship between the *APOLIPOPROTEIN* (*APO)E* genotype and cardiovascular disease (CVD) risk. Using data from the BODYCON cross-sectional study (n = 360 adults) we assessed the association between body composition and CVD risk markers according to *APOE* genotype, with examination of the role of BMI. In this study cohort, the *APOE2/E3* group had lower fasting blood lipids than *APOE4* carriers and *APOE3/E3* group (*p* ≤ 0.01). After stratifying the group according to BMI, *APOE4* carriers in the normal BMI subgroup had a higher lean mass compared with the *APOE3/E3* group (*p* = 0.02) whereas in the overweight/obese subgroup, the android to gynoid percentage fat ratio was lower in *APOE4* carriers than *APOE3/E3* group (*p* = 0.04). Fasting lipid concentrations were only different between the *APOE2/E3* and other genotype groups within the normal weight BMI subgroup (*p* ≤ 0.04). This finding was associated with a lower dietary fibre and a higher trans-fat intake compared with *APOE4* carriers, and a lower carbohydrate intake relative to the *APOE3/E3* group. Our results confirm previous reports that BMI modulates the effect of *APOE* on CVD risk markers and suggest novel interactions on body composition, with diet a potential modulator of this relationship.

## 1. Introduction

The *APOLIPOPROTEIN (APO)E* gene is one of the most widely studied in relation to cardiovascular disease (CVD) risk due to the association with circulating blood lipids. It encodes the multifunctional apoE apoprotein which represents an important ligand for the receptor-mediated uptake of triacylglycerol (TAG)-rich lipoproteins and their remnants from the circulation [1]. The *APOE2*, *APOE3* and *APOE4* alleles have different affinities for the low-density lipoprotein (LDL) receptor which impacts on cholesterol homeostasis and blood lipid profile [2,3,4]. It has been well documented that the *APOE* gene accounts for 7% of the variance in cholesterol in Caucasians [5]. Although several studies have reported elevated total (TC) and LDL cholesterol (LDL-C) concentrations in *APOE4* carriers and lower concentrations in *APOE2* carriers compared to the wild-type *APOE3/E3* group [6,7], these relationships have not been reported by others [8,9,10,11,12]. These inconsistencies between studies have been attributed to the metabolic status and adiposity of the study populations suggesting that other factors such as body mass index (BMI) may impact on the relationship between the *APOE* genotype and chronic disease risk [10,11,12].

It is well-known that obesity is an independent risk factor for CVD [13]. Animal studies have shown apoE knock out mice to be protected against obesity [14,15] and suggested a differential effect of the *APOE* alleles on the ability of the body to store fat, with *APOE3* mice having a higher body weight than *APOE4* mice on a Western type diet [16,17,18]. Moreover, increased visceral adipose tissue (VAT) accumulation, which is associated with increased CVD risk [19,20], was reported in *APOE3* compared to *APOE4* mice [17,21,22]. Therefore, a possible explanation for the inconsistent results on association between the *APOE* genotype and blood cholesterol concentrations may be dependent on adiposity. In agreement, several studies have reported the relationship between *APOE* and blood lipid risk markers to differ depending on BMI but the mechanisms underlying this association are unclear. Lower TAG concentrations in *APOE2* carriers compared with the *APOE3/E3* group and *APOE4* carriers was reported to be evident only in the UK adults with a normal BMI [23], whereas in Mexican Amerindian population, differences in TC, LDL-C and TAG among *APOE4* carriers and *APOE3/E3* genotype group were only found in obese subjects (BMI ≥ 30 kg/m^2^) [24]. However, the limited human studies conducted to date have failed to identify which *APOE* allele is more prone to obesity and whether an interaction exists between *APOE* and adiposity on CVD risk markers [23,24,25,26,27]. In addition, the *APOE* genotype may have an impact on food preferences which can affect body composition. However, the evidence is limited [28].

Therefore, this paper aims to investigate the association between the *APOE* genotype with body composition and CVD risk markers, with further examination of the role of BMI on this relationship.

## 2. Results

### 2.1. The Effect of APOE Genotype on Body Composition Measures and Cardiovascular Disease Risk Markers

The main characteristics for 360 participants (187 female and 168 male) according to the *APOE* genotype is shown in Table 1 and presented for women and men separately in Supplementary Tables S1 and S2, respectively. The study population had an average age of 42 ± 1 y and BMI of 24.1 ± 0.2 kg/m^2^, and n = 46 participants were *APOE2/E3*, n = 228 the wild type *APOE3/E3* group and n = 81 *APOE4* carriers (*APOE3/E4* and *APOE4/E4*). Subjects with the *APOE2/E4* genotype (n = 5) were not included in the analysis due to the small sample size and no participants with the *APOE2/E2* genotype were identified in the study cohort. The *APOE* allele distribution was found to be in Hardy–Weinberg equilibrium.

In the group as a whole, fasting TC (*p* = 0.01), LDL-C (*p* ≤ 0.01) and non-high density lipoprotein cholesterol (HDL-C) concentrations (*p* ≤ 0.01) and LDL-C:HDL-C ratio (*p* = 0.02) in the *APOE2/E3* group were on average 9–18% lower compared to *APOE4* carriers and 9–16% lower compared with the *APOE3/3* group (Table 1). Although genotype was found to have a significant effect on diastolic blood pressure (*p* = 0.04), differences between the genotype groups was not evident after post hoc analysis. Furthermore, anthropometric and body composition measures were not different between the genotype groups. The habitual dietary intakes of participants are shown in Table 1. Total dietary fibre intake was on average 4 g higher in *APOE4* carriers than *APOE2/E3* group (*p* = 0.04), while there was no difference in intake in the *APOE3/E3* group compared to the other genotype groups. There was also an influence of *APOE* on total protein intake, with the *APOE3/E3* group consuming 3% of total energy (TE) (approximately 5 g) lower than participants in the *APOE2/E3* group (*p* < 0.01), but the intake in the *APOE4* carriers was not different to the other genotype groups. The *APOE* genotype did not affect total dietary energy or intake of other macronutrients.

After stratifying the group according to sex, a few differences were evident on the CVD risk markers and dietary intake according to *APOE* genotype in men and women.

Genotype had a significant impact on blood lipids, dietary energy intake and total carbohydrate and protein intakes in women whereas in men, the effect was on diastolic blood pressure and NEFA. In women, the LDL-C and non-HDL-C concentrations and LDL-C:HDL-C ratio were significantly lower in *APOE2/E3* group compared to the *APOE3/E3* group and *APOE4* carriers (*p* < 0.01 for each). Dietary energy intake (*p* = 0.05) was lower in the *APOE2/E3* group compared with *APOE4* carriers only and was associated with a significantly lower carbohydrate intake (*p* = 0.02) compared with the *APOE3/E3* group but higher total protein intake (*p* < 0.01) relative to both the *APOE3/E3* and *APOE4* carrier groups (Supplementary Table S1). In men, diastolic blood pressure and the fasting NEFA concentration were lower in the *APOE4* carriers compared to the *APOE3/E3* but not *APOE2/E3* group (*p* < 0.01 for each) (Supplementary Table S2).

### 2.2. Effect of APOE Genotype and BMI on Body Composition Measures and CVD Risk Markers

Significant genotype x BMI interactions were observed for android fat mass (a measure of central adiposity) and for the dietary intakes of total polyunsaturated fatty acids (%TE) and total protein (%TE) (*p* ≤ 0.03) only. There was no significant impact on other measures of body composition, dietary intakes, or CVD risk markers (Table 1).

To assess the effect of the *APOE* genotype according to BMI, participants were split into normal weight (BMI ≤ 24.9 kg/m^2^, n = 232) and overweight/obese (BMI ≥ 25 kg/m^2^, n = 128) subgroups. The subject characteristics, body composition and CVD risk markers according to the BMI subgroups are shown in Table 2. In the normal weight BMI subgroup, *APOE4* carriers had on average a 3 kg higher lean mass (*p* = 0.02) and 0.24 kg greater android lean mass (*p* = 0.01) than the wild-type *APOE3/E3* group, while there was no difference in lean mass or android lean mass in the *APOE2/E3* group compared to the other genotype groups. Fasting LDL-C (*p* = 0.01) and non-HDL-C (*p* = 0.02) concentrations were 17% and 15% lower, respectively in the *APOE2/E3* group compared to the *APOE4* carriers and 15% and 12% lower compared to the *APOE3/E3* group. The LDL-C:HDL-C ratio was also 17% lower in the *APOE2/E3* group compared to *APOE4* carriers (*p* = 0.04), with no differences found between the *APOE3/E3* group compared to *APOE2/E3* and *APOE4* carriers. TC concentrations were 9% lower in the *APOE2/E3* compared with the *APOE3/E3* group (*p* = 0.04), but not in the *APOE4* carriers. In the overweight/obese BMI group, the android to gynoid percentage fat ratio was higher in the *APOE3/E3* group compared to *APOE4* carriers (*p* = 0.04), while there was no difference in the *APOE2/E3* compared to the other genotype groups. Other body composition measures and CVD risk factors did not differ across the three genotype groups in this BMI subgroup (Table 2).

Habitual dietary intakes are presented in Table 3 according to normal and overweight/obese BMI subgroups. In the normal BMI group, while dietary fibre intake (*p* = 0.02) was 6 g higher, trans-fat (%TE) (*p* = 0.05) was 0.15% (5.7 g) lower in *APOE4* carriers compared to the *APOE2/E3* group, with no differences found compared with the *APOE3/E3* group. The participants in the *APOE2/E3* group also had a lower dietary carbohydrate (%TE) intake compared to the *APOE3/E3* group (*p* = 0.01) only. Moreover, in the normal BMI subgroup, the *APOE2/E3* group had the highest total protein (%TE) intake compared to the *APOE3/E3* group (*p* = 0.01), while the carbohydrate intake of the *APOE4* carriers was not different versus the *APOE2/E3* and *APOE3/E3* groups. Dietary intakes were not different between genotype groups in the overweight/obese BMI group (Table 3). Physical activity levels (steps/day, energy expended performing physical activity per day, and percentage time spent performing sedentary, light, or moderate to vigorous physical activity) were not significantly different according to *APOE* genotype neither in the whole group or after stratifying according to normal and overweight/obese BMI subgroups (Supplementary Tables S3 and S4).

## 3. Discussion

This study examined the association between the *APOE* genotype with body composition and CVD risk factors and the impact of BMI classification on this relationship. Using data from the impact of physiological and lifestyle factors on body composition (BODYCON) cross-sectional study, we found the *APOE* genotype to impact on the fasting lipid profile, with differences only evident in participants with a normal BMI and in women. Novel associations between genotype and body composition were observed, with divergent effects of *APOE* on the android to gynoid percentage fat ratio (an estimate of body fat distribution) and lean body mass within the normal and overweight/obese BMI subgroups.

Several studies have reported associations between the *APOE* genotype and fasting blood lipid risk markers [29]. In agreement with the previous studies [6,7,30], we also observed TC, LDL-C and non-HDL-C concentrations to be significantly higher in *APOE4* carriers and *APOE3/E3* group compared to the *APOE2/E3* group. However, after dividing the cohort into BMI subgroups, LDL-C and non-HDL-C concentrations were only significantly higher in *APOE4* carrier and *APOE3/E3* groups compared to the *APOE2/E3* group in the normal BMI subgroup. Our findings support those of Kofler et al. [23] who reported the lowest fasting TAG concentration in *APOE2* carriers only in participants with normal BMI in the FINGEN study where 312 participants living in the UK were prospectively genotyped for *APOE*. In agreement with this, Kolovou et al. [31] observed the *APOE4* allele to be associated with higher TC levels compared with *APOE3* allele in normal-weight coronary heart disease patients based in Greece. Although not widely studied [32,33], we also observed sex-dependent effects on the fasting lipid profile, with differences only evident between genotype groups in the women but not men. Of note, BMI was also significantly lower in the women compared with the men [34]. Therefore, our data shows that the effect of *APOE* on CVD risk markers may be dependent on their BMI and that the negative metabolic effects of high BMI could mask the effect of the *APOE* genotype on the fasting lipid profile. For example, our findings show that the detrimental effect of an increased BMI outweighs the positive effect of the *APOE2* allele on blood lipid risk markers. Diet and physical activity levels are important modifiable determinants of BMI [35]. It should be noted that *APOE4* carriers consumed more dietary fibre and less trans-fat compared to *APOE2* carriers. Similarly, studies have shown an impact of the *APOE* genotype on protein intake. For example, in the Australian Imaging, Biomarkers and Lifestyle study of ageing *APOE4* carriers were found to have lower protein intakes than non-*APOE4* carriers [19]. Therefore, our study may lend support to the findings that *APOE* has an impact on food preferences which can affect body composition. Furthermore, our study included healthy subjects with a higher-than-average physical activity level. This may have impacted on the fasting lipid profile observed within the genotype groups as exercise has been shown to favourably affect total cholesterol and TAG levels [36]. Thus, further studies are needed examining the role of *APOE* genotype on food intake and relationship to BMI.

The effect of *APOE* genotype on body composition has been investigated in animals and a small number of human studies. Arbones-Mainar et al. [17] reported greater increases in abdominal VAT accumulation and body weight after a high fat western type diet (21%TE fat) in *APOE3* mice compared to *APOE4*. Although our participants consumed on average a high fat diet (37%TE), abdominal VAT was not different between the *APOE* genotype groups. However, findings from human studies investigating the association between the *APOE* genotype and body composition are inconsistent. Positive associations between the *APOE2* allele with waist circumference and BMI have been reported in 230 Croatian subjects aged 20–85 y and in 4660 Caucasian middle-aged men [37,38]. Another study in 290 children aged 8 years reported lower BMI, trunk fat mass and waist circumference in *APOE4* carriers compared to non-*APOE4* carriers (*APOE3/E3*, *APOE2/E2* and *APOE2/E3*) [39]. In contrast, in a case–control study including 198 normal weight healthy and 198 obese Saudi university students, the *APOE4* allele was positively associated with BMI in overweight and obese subjects (BMI > 25 kg/m^2^) [40]. These discrepancies between studies might be influenced by the participants sex, age, ethnicity and/or habitual diet. Therefore, it is important to take these factors into consideration when comparing study findings.

In the current study, we found a genotype x BMI interaction on android fat mass. After stratifying the cohort according to BMI, there were no differences in body weight between *APOE* genotypes in either the normal or overweight/obese BMI subgroups. However, we observed *APOE4* carriers with a normal BMI to have higher lean body mass and android lean mass compared to the wild-type *APOE3/E3* group. Therefore, this might provide a possible explanation for the lower body fat and VAT mass accumulation in the *APOE4* carriers compared to the *APOE3/E3* group in animal studies [16,21]. The mechanisms behind the relationship between the *APOE4* allele and increased lean body mass are not clear although animal studies have suggested that adiponectin may play a role. In *APOE4* mice, a greater increase in adiponectin levels were observed compared to *APOE3* mice on an obesogenic diet [41] and the protective role of adiponectin against muscle loss and muscle growth have been described in some studies [42]. In addition, an association between appendicular lean mass (lean tissue in arms and legs) and circulating adiponectin was reported in postmenopausal women [43]. However, in this study adiponectin concentrations were not different between the genotype groups, therefore this potential mechanism needs to be examined in further studies. Moreover, in the overweight/obese BMI subgroup *APOE4* carriers had a lower android to gynoid fat percentage ratio suggesting a difference in body fat distribution compared to the wild-type group which had similar dietary intakes and physical activity levels. These findings are interesting since it is well-known that abdominal obesity is associated with dyslipidaemia [44], and our findings suggest that the *APOE3/E3* genotype group had lower LDL-C and non-HDL-C concentrations, but higher android body fat distribution compared to *APOE4* carriers. Our finding provide support to those of a previous study which reported that *APOE4* mice accumulated less VAT than *APOE3* mice after following a high fat diet for 6 months [18]. The authors speculated that endoplasmic reticulum stress is a potential mechanism linking *APOE* and adiposity. Since apoE4 has a lower protein stability and is abnormally folded in the endoplasmic reticulum, increased endoplasmic reticulum stress in *APOE4* carriers may have a negative effect on adipogenesis [18]. Moreover, in a study by Huebbe et al. [16] less weight gain in *APOE4* compared to *APOE3* mice on high and low-fat diets was observed and the authors reported higher expression of fatty acid-binding protein 4, carnitine palmitoyl transferase 1B and uncoupling protein in *APOE4* mice which suggested increased fatty acid oxidation in skeletal muscle in *APOE4* mice compared to the *APOE3* mice. However, as mice do not usually consume high fat diets it is difficult to translate findings from animal studies to humans. Therefore, further clarification of the association between *APOE* and body fat distribution measures and the potential mechanisms observed are needed in humans.

The use of a dual energy X-ray absorptiometry (DXA) which is known to be an accurate and precise tool for body composition measurement and includes an estimation of abdominal VAT mass is an important strength of this study. In addition, we included the analysis of a range of outcome measures such as physical activity, dietary intake and CVD risk markers in this cohort. Limitations include the cross-sectional study design, retrospective genotyping and small sample size for some genotype groups, especially during the sub-group analysis according to BMI and sex. Moreover, subjects were not stratified according to the median BMI but as normal and overweight/obese subgroups for translation of our findings to UK public health guidance, with only 36% of this cohort having a BMI > 24.9 kg/m^2^. Finally, it should be noted that using BMI as a marker of adiposity to stratify the group has its own limitations since it cannot distinguish between excess body fat and muscle mass.

In summary, our results indicate an interaction between the *APOE* genotype and BMI, with higher fasting lipid risk marker concentrations only evident in *APOE4* carriers compared to the *APOE2/E3* group in participants with a normal BMI and in women. Moreover, differential effects on body fat distribution and composition were observed within the BMI subgroups between the *APOE4* carriers and the wild-type *APOE3/E3* group, with diet also a potential modulator of this relationship. However, the association between *APOE* genotype, adiposity, sex, diet and CVD risk markers needs further investigation in humans with prospective genotyping to draw a firm conclusion.

## 4. Materials and Methods

### 4.1. Subjects

A total of 360 healthy men and women aged 18–70 y from the BODYCON study were included in the present analysis. Details of the study design have been described previously [34]. Briefly, participants were recruited from Reading and the surrounding areas and inclusion criteria were BMI 18.5–39.9 kg/m^2^, TC < 7.8 mmol/L, TAG < 2.3 mmol/L, fasting blood glucose < 7.8 mmol/L, haemoglobin > 115 g/L for women and 130 g/L for men. Exclusion criteria were having suffered a myocardial infarction/stroke in the past 12 months, history of diabetes or other endocrine disorders, bowel disease, cholestatic liver disease, pancreatitis, cancer, arthritis or fracture deformity of spine or femur, history of bone related surgeries, radio-opaque implants or implanted medical devices, breastfeeding, being pregnant or planning pregnancy in the next 12 months, being a smoker, being on medication for hyperlipidemia, hypertension, inflammation or hypercoagulation, being on a weight reducing diet and excessive alcohol consumption (<14 units/wk). Female subjects taking oral contraceptives or HRT for at least 3 months were included in the study.

### 4.2. Study Design

The BODYCON study is an observational cross-sectional study conducted in the Hugh Sinclair Unit of Human Nutrition at the University of Reading. The main outcomes of the BODYCON study have been described previously [34]. Briefly, participants attended a single study visit in which a fasting blood sample was collected, and anthropometric measurements were taken. Participants also underwent a DXA scan to assess their total body composition. The NHS and University of Reading Research Ethics Committees both gave a favourable ethical opinion for the conduct of the BODYCON study (NHS reference number: 14/SC/1095 and UREC reference numbers: 17/29 and 13/55). Participants were only included in the current analysis if written consent was obtained for the retrospective genotyping for *APOE*. The BODYCON study was carried out in accordance with the principles of the Declaration of Helsinki and registered at www.clinicaltrials.gov (accessed on 1 January 2022) (NCT02658539).

### 4.3. Anthropometric Measurements

Anthropometric measures were performed with participants wearing light clothing and no shoes. Height was measured by a stadiometer. Body weight and BMI were determined using a Tanita BC-418 scale (TANITA UK Ltd., Middlesex, UK). Waist and hip circumferences were measured using a non-stretch tape measure. To assess the body composition, DXA scan was performed by trained researchers and described elsewhere [34]. Briefly, prior to the scan participants were required to wear clothes without metal fastenings, buttons or zips, and all metal artefacts were removed. For the total body composition scan, participants lay still on the Lunar iDXA scanner bed with Velcro straps around their knees and ankles. All scans were analysed using enCORE Software, version 15 (GE Medical Systems Ltd, Chalfont St Giles, UK) with the advance software package CoreScan, which also estimates the mass and volume of VAT within the abdomen.

### 4.4. Dietary Intakes and Physical Activity

Habitual dietary intake was assessed using a 4-day weighed diet diary. Dietary data was analysed using DietPlan 7 software (Forestfield, Horsham, UK) and dietary intakes were averaged. Participants with dietary intakes outside of the ranges 500–3500 kcal for women and 800–4000 kcal for men were reported to be under/over reporters (n = 4) and excluded from the dietary analysis [45]. Participants with competition of <3 days of diet diary (n = 1) were also excluded from the dietary analysis. Physical activity levels were measured using a tri-axial accelerometer (Actigraph wGT3X+, Actigraph LLC, Pensacola, FL, USA). Participants were asked to wear the accelerometer directly above the right iliac crest during sleeping and waking hours (except for during water-based activities) for four days, including three week days and one weekend day during the same time that dietary intake was assessed. Device initialization, data processing and analysis were conducted using Actilife Data Analysis Software (Version 6.11.5).

### 4.5. Biochemical Analysis

Fasting blood samples collected into the serum separator and K_3_EDTA blood tubes were centrifuged at 1700× *g* (3000 rpm) for 15 min at room temperature and 4 °C, respectively before aliquoting into Eppendorf tubes and stored at −20 °C. Fasting lipids (TC, HDL-C, non-esterified fatty acids (NEFA), TAG), glucose and high sensitivity C-reactive protein (hs-CRP)) were quantified in the serum sample by using the ILAB 600 (Werfen (UK) Ltd., Warrington, UK) and RX Daytona Plus (Randox Laboratories Limited, Crumlin, UK) clinical chemistry analysers. The Friedewald equation was used to estimate fasting LDL-C concentrations [46] and non-HDL-C was calculated by subtracting HDL-C from TC. Plasma uric acid was measured using Daytona Plus clinical chemistry analyser (Randox Laboratories Ltd., Crumlin, UK). ELISA kits were used to analyse serum insulin (Dako UK Ltd. Ely, UK and Crystal Chem, Inc., Elk Grove Village, IL, USA) and plasma adiponectin (Quantikine kit, R&D Systems, Europe Ltd, Abingdon, UK.).

### 4.6. DNA Extraction and Genotyping

The buffy coat layer was isolated from the blood sample collected into a 9 mL EDTA blood tube prior to the extraction of DNA using a DNA blood mini kit (Qiagen Ltd., Manchester, UK) according to the manufacturers protocol. DNA samples were genotyped for the single nucleotide polymorphisms (SNP) rs429358 and rs7412 with the use of TaqMan SNP genotyping assays on the QuantStudio 3 real time PCR machine (Life Technologies Limited, Paisley, UK).

### 4.7. Statistical Analysis

Statistical analyses were performed using IBM SPSS Statistics version 25 (SPSS Inc., Chicago, IL, USA). Normality of data was checked using Kolmogrov-Smirnov test and Q-Q plots. Hardy–Weinberg equilibrium was tested by a chi-square test. To assess the effect of the *APOE* genotype and BMI, a general linear model (ANCOVA) was performed using the study outcome measures as the dependent variable, genotype or BMI as a fixed factor and age and sex as covariates. The effect of *APOE* according to sex was assessed by a general linear model (ANCOVA) using the study outcome measures as the dependent variable, genotype as a fixed factor and age as a covariate after splitting the data according to sex. To assess the effect of adiposity, a BMI x genotype interaction was added to the model. Participants were then stratified into normal and overweight/obese BMI subgroups and analysed using ANCOVA including age and sex as covariates. If a significant genotype effect was found, pairwise comparisons with a Bonferroni correction were carried out. Results are presented as estimated marginal means ± SE and *p* ≤ 0.05 was considered significant.

## Figures and Tables

**Table 1:** Participant characteristics including anthropometric measures, CVD risk markers and habitual dietary intakes according to *APOE* genotype ^1^.

	*APOE* Genotype				*p*		
	All (n = 360)	*E2* Carriers (n = 46)	*E3/E3* (n = 228)	*E4* Carriers (n = 81)	Genotype ^2^	BMI ^2^	Genotype × BMI ^3^
Frequency (%)	-	12.8	63.3	22.5			
Sex, F/M	187/168	28/18	121/107	38/43			
Age (y)	42 ± 1	45 ± 2	41 ± 1	44 ± 2	0.23		
BMI (kg/m^2^)	24.1 ± 0.2	23.7 ± 0.5	24.2 ± 0.2	24.2 ± 0.4	0.58		
**Anthropometric and Body Composition Measurements**							
WC (cm)	84.3 ± 0.6	83.8 ± 1.5	84.6 ± 0.7	84.0 ± 1.1	0.83	<0.01	0.12
HC (cm)	101 ± 1	100 ± 1	101 ± 1	102 ± 1	0.52	<0.01	0.72
Body fat (%)	28.2 ± 0.4	28.1 ± 1.0	28.4 ± 0.5	27.9 ± 0.8	0.83	<0.01	0.60
Fat mass (kg)	20.3 ± 0.4	19.6 ± 1.2	20.5 ± 0.5	20.4 ± 0.9	0.78	<0.01	0.42
Lean mass (kg)	48.5 ± 0.6	48.0 ± 1.0	48.4 ± 0.4	49.4 ± 0.7	0.36	<0.01	0.21
Abdominal VAT (g)	599 ± 31	561 ± 70	622 ± 32	569 ± 53	0.57	<0.01	0.70
Android fat mass (kg)	1.61 ± 0.05	1.52 ± 0.14	1.65 ± 0.07	1.57 ± 0.11	0.63	<0.01	<0.01
Android lean mass (kg)	3.32 ± 0.04	3.27 ± 0.07	3.30 ± 0.03	3.43 ± 0.05	0.06	0.01	0.39
Android fat (%)	30.5 ± 0.6	30.4 ± 1.7	30.9 ± 0.8	29.6 ± 1.3	0.65	<0.01	0.74
Gynoid fat (%)	32.1 ± 0.5	31.8 ± 1.0	32.2 ± 0.5	32.1 ± 0.8	0.94	<0.01	0.42
A/G fat % ratio	0.96 ± 0.02	0.98 ± 0.03	0.97 ± 0.02	0.92 ± 0.03	0.26	<0.01	0.87
**Cardiovascular Disease Risk Markers**							
Blood pressure (mmHg)							
Systolic	120 ± 1	118 ± 2	121 ± 1	119 ± 1	0.35	0.40	0.11
Diastolic	72 ± 1	69 ± 1	73 ± 1	71 ± 1	0.04	0.24	0.96
Pulse pressure	48 ± 1	49 ± 2	48 ± 1	48 ± 1	0.96	0.47	0.06
TC (mmol/L)	5.16 ± 0.06	4.77 ± 0.14 ^b^	5.22 ± 0.06 ^a^	5.25 ± 0.10 ^a^	0.01	0.22	0.90
TAG (mmol/L)	0.98 ± 0.03	1.02 ± 0.07	0.96 ± 0.03	1.00 ± 0.05	0.63	<0.01	0.80
HDL-C (mmol/L)	1.65 ± 0.02	1.68 ± 0.05	1.66 ± 0.02	1.61 ± 0.04	0.42	0.20	0.97
LDL-C (mmol/L)	3.05 ± 0.05	2.63 ± 0.12 ^b^	3.11 ± 0.05 ^a^	3.18 ± 0.09 ^a^	<0.01	0.28	0.87
Non-HDL-C (mmol/L)	3.51 ± 0.05	3.09 ± 0.13 ^b^	3.56 ± 0.06 ^a^	3.64 ± 0.10 ^a^	<0.01	0.11	0.93
TC:HDL-C ratio	3.25 ± 0.05	3.01 ± 0.12	3.27 ± 0.06	3.35 ± 0.09	0.09	<0.01	0.99
LDL-C:HDL-C ratio	1.94 ± 0.04	1.69 ± 0.11 ^b^	1.97 ± 0.05 ^a^	2.05 ± 0.08 ^a^	0.02	0.01	0.99
NEFA(μmol/L)	398 ± 12	404 ± 32	390 ± 14	417 ± 24	0.61	0.11	0.19
Glucose (mmol/L)	5.04 ± 0.03	5.00 ± 0.07	5.04 ± 0.03	5.03 ± 0.05	0.88	0.04	0.47
CRP (mg/L)	1.35 ± 0.12	1.01 ± 0.34	1.48 ± 0.15	1.24 ± 0.26	0.40	0.43	1.00
Adiponectin (µg/mL)	6.55 ± 0.29	5.28 ± 0.76	6.69 ± 0.35	6.58 ± 0.57	0.24	0.88	0.74
Uric acid (µmol/L)	275 ± 4	286 ± 8	275 ± 4	272 ± 6	0.37	0.38	0.64
**Dietary Intake**							
Energy intake (MJ)	8.5 ± 0.2	7.8 ± 0.3	8.6 ± 0.2	8.7 ± 0.3	0.06	0.58	0.57
Total fat (%TE)	36.6 ± 0.5	37.5 ± 1.3	36.3 ± 0.6	36.6 ± 1.0	0.68	0.12	0.79
SFA (%TE)	13.0 ± 0.2	13.4 ± 0.7	13.0 ± 0.3	12.6 ± 0.5	0.67	0.10	0.90
MUFA (%TE)	13.7 ± 0.2	13.9 ± 0.6	13.6 ± 0.3	13.9 ± 0.4	0.80	0.14	0.57
PUFA (%TE)	6.3 ± 0.1	6.3 ± 0.3	6.1 ± 0.2	6.7 ± 0.3	0.08	0.76	0.05
Trans fat (%TE)	0.55 ± 0.02	0.59 ± 0.04	0.54 ± 0.02	0.53 ± 0.03	0.55	0.05	0.27
Total CHO (%TE)	45.4 ± 0.6	42.5 ± 1.7	46.5 ± 0.8	44.8 ± 1.3	0.07	0.37	0.79
Total sugars (%TE)	18.5 ± 0.4	17.5 ± 1.0	18.6 ± 0.5	19.1 ± 0.8	0.43	0.56	0.23
Total fibre (AOAC, g)	24.6 ± 0.5	22.3 ± 1.3 ^b^	24.4 ± 0.6 ^ab^	26.7 ± 1.0 ^a^	0.03	0.94	0.45
Total protein (%TE)	18.4 ± 0.3	20.9 ± 0.8 ^b^	17.9 ± 0.4 ^a^	18.7 ± 0.6 ^ab^	<0.01	0.91	<0.01

^1^ Data was presented as estimated marginal means ± SE, *p* < 0.05 is considered significant. *E2* carriers = *E2*/*E3*, *E4* carriers = *E3*/*E4* and *E4*/*E4*. ^2^ Data was analysed by univariate general linear model (ANCOVA) adjusted for age and sex. ^3^ *APOE* genotype × BMI interaction by ANCOVA, adjusted for age and sex. Carrier code and BMI as fixed factors and variable of interest as dependent variable. ^ab^ significant differences (*p* < 0.05) shown as different superscript letters. Sample sizes are as follows: for WC, HC, all n = 359, *E2* carriers n = 46, *E3/E3* n = 227, *E4* carriers n = 81; for Blood Pressure, all n = 357, *E2* carriers n = 46, *E3/E3* n = 225, *E4* carriers n = 81; for NEFA all n = 355 *E2* carriers n = 45, *E3/E3* n = 225, *E4* carriers n = 80; for CRP all n = 359, *E2* carriers n = 46, *E3/E3* n = 227, *E4* carriers n = 81; for Adiponectin and Uric acid all n = 322 *E2* carriers n = 42, *E3/E3* n = 201, *E4* carriers n = 75, Dietary Intakes all n = 345 *E2* carriers n = 44, *E3/E3* n = 219, *E4* carriers n = 77. Abbreviations: AOAC—Association of official analytical chemists, A/G fat % ratio—android to gynoid fat % ratio, BMI—body mass index, CHO—carbohydrate, CRP—C-reactive protein, HC—hip circumference, HDL-C—high-density lipoprotein cholesterol, LDL-C—low-density lipoprotein cholesterol, MUFA—monounsaturated fatty acids, NEFA—non-esterified fatty acids, PUFA—polyunsaturated fatty acids, SFA—saturated fatty acids, TC—total cholesterol, TAG—triacylglycerol, VAT—visceral adipose tissue, WC—waist circumference.

**Table 2:** Participant characteristics and anthropometric measures according to *APOE* genotype in normal and overweight/obese BMI subgroups.

	BMI ≤24.9 kg/m^2^ (n = 232)				BMI ≥25 kg/m^2^ (n = 128)			
	*E2* Carriers (n = 33)	*E3/E3* (n = 147)	*E4* Carriers (n = 48)	*p*	*E2* Carriers (n = 13)	*E3/E3* (n = 81)	*E4* Carriers (n = 33)	*p*
Frequency (%)	14.2	63.4	20.7		10.2	63.3	25.8	
Female/male	22/11	85/62	26/22		6/7	36/45	12/21	
Age (y)	43 ± 3	40 ± 1	44 ± 2	0.26	50 ± 4	44 ± 2	44 ± 2	0.40
**Anthropometric and Body Composition Measurements**								
Weight (kg)	65.0 ± 1.2	63.1 ± 0.6	65.4 ± 1.0	0.08	80.8 ± 2.7	84.7 ± 1.1	82.1 ± 1.7	0.25
BMI	22.3 ± 0.3	22.0 ± 0.1	22.0 ± 0.3	0.64	27.2 ± 0.8	28.3 ± 0.3	27.7 ± 0.5	0.33
WC (cm)	80.1 ± 1.1	78.3 ± 0.5	78.0 ± 0.9	0.28	92.4 ± 2.4	95.6 ± 1.0	93.6 ± 1.5	0.32
HC (cm)	98.2 ± 1.0	96.6 ± 0.5	97.8 ± 0.9	0.24	106.3 ± 2.1	109.4 ± 0.8	108.7 ± 1.3	0.38
Body fat (%)	26.5 ± 0.9	25.6 ± 0.4	23.8 ± 0.8	0.06	32.3 ± 1.6	33.3 ± 0.7	33.9 ± 1.0	0.70
Fat mass (kg)	17.1 ± 0.7	16.1 ± 0.3	15.5 ± 0.6	0.20	26.0 ± 2.0	28.2 ± 0.8	28.0 ± 1.2	0.57
Lean mass (kg)	46.1 ± 1.0 ^ab^	45.2 ± 0.5 ^b^	48.1 ± 0.9 ^a^	0.02	52.4 ± 1.7	54.0 ± 0.7	51.8 ± 1.1	0.20
Abdominal VAT (g)	380 ± 44	341 ± 21	331 ± 36	0.66	929 ± 138	1126 ± 55	963 ± 86	0.17
Android fat mass (kg)	1.21 ± 0.12	1.11 ± 0.06	1.01 ± 0.10	0.42	2.25 ± 0.23	2.61 ± 0.09	2.44 ± 0.15	0.27
Android lean mass (kg)	3.15 ± 0.01 ^ab^	3.10 ± 0.04 ^b^	3.34 ± 0.07 ^a^	0.01	3.54 ± 0.13	3.65 ± 0.05	3.60 ± 0.08	0.66
Android fat (%)	27.2 ± 1.5	25.2 ± 0.7	22.9 ± 1.3	0.09	38.2 ± 2.5	41.1 ± 1.0	39.8 ± 1.5	0.47
Gynoid fat (%)	30.7 ± 1.0	30.4 ± 0.5	28.5 ± 0.8	0.11	35.2 ± 1.7	35.5 ± 0.7	37.4 ± 1.1	0.29
A/G fat % ratio	0.90 ± 0.03	0.85 ± 0.02	0.82 ± 0.03	0.18	1.14 ± 0.05 ^ab^	1.19 ± 0.02 ^b^	1.10 ± 0.03 ^a^	0.04
**Cardiovascular Disease Risk Markers**								
Blood pressure (mmHg)								
Systolic	116 ± 2	119 ± 1	118 ± 2	0.67	123 ± 3	125 ± 1	122 ± 2	0.48
Diastolic	68 ± 2	71 ± 1	70 ± 1	0.19	73 ± 3	76 ± 1	73 ± 1	0.16
Pulse pressure	49 ± 2	48 ± 1	48 ± 2	0.94	49 ± 3	49 ± 1	49 ± 2	0.99
TC (mmol/L)	4.70 ± 0.17 ^b^	5.14 ± 0.08 ^a^	5.19 ± 0.14 ^ab^	0.04	4.91 ± 0.26	5.35 ± 0.10	5.34 ± 0.16	0.27
TAG (mmol/L)	0.88 ± 0.06	0.82 ± 0.03	0.90 ± 0.05	0.29	1.32 ± 0.16	1.20 ± 0.06	1.17 ± 0.10	0.72
HDL-C (mmol/L)	1.72 ± 0.06	1.76 ± 0.03	1.68 ± 0.05	0.40	1.58 ± 0.09	1.49 ± 0.04	1.49 ± 0.06	0.66
Non-HDL-C (mmol/L)	2.98 ± 0.15 ^b^	3.39 ± 0.07 ^a^	3.51 ± 0.12 ^a^	0.02	3.33 ± 0.25	3.87 ± 0.10	3.85 ± 0.16	0.14
LDL-C (mmol/L)	2.57 ± 0.14 ^b^	3.01 ± 0.07 ^a^	3.10 ± 0.12 ^a^	0.01	2.73 ± 0.23	3.28 ± 0.09	3.32 ± 0.14	0.07
NEFA (μmol/L)	402 ± 39	405 ± 19	423 ± 33	0.88	420 ± 56	361 ± 22	406 ± 34	0.41
TC:HDL-C ratio	2.83 ± 0.11	2.99 ± 0.05	3.14 ± 0.09	0.07	3.42 ± 0.29	3.77 ± 0.11	3.68 ± 0.18	0.52
LDL-C:HDL-C ratio	1.57 ± 0.10 ^b^	1.76 ± 0.05 ^ab^	1.89 ± 0.08 ^a^	0.04	1.93 ± 0.25	2.34 ± 0.10	2.30 ± 0.15	0.29
Glucose (mmol/L)	5.00 ± 0.08	4.95 ± 0.04	5.00 ± 0.07	0.68	5.00 ± 0.12	5.21 ± 0.05	5.08 ± 0.08	0.14
CRP (mg/L)	0.83 ± 0.43	1.31 ± 0.20	0.88 ± 0.36	0.42	1.47 ± 0.56	1.78 ± 0.23	1.80 ± 0.35	0.87
Adiponectin (µg/mL)	5.18 ± 0.97	7.30 ± 0.46	6.38 ± 0.79	0.12	5.53 ± 1.20	5.48 ± 0.51	6.77 ± 0.76	0.36
Uric acid(µmol/L)	271 ± 9	269 ± 4	254 ± 7	0.16	322 ± 17	286 ± 7	302 ± 11	0.13

Data was presented as estimated marginal means ± SE, *p* < 0.05 is considered significant *E2* carriers = *E2/E3*, *E4* carriers = *E3/E4* and *E4/E4*. Data was analysed by univariate general linear model (ANCOVA) adjusted for age and sex. ^ab^ significant differences (*p* < 0.05) shown as different superscript letters. Sample sizes are as follows: WC, HC BMI ≤ 24.9 kg/m^2^; *E2* carriers n = 33, *E3/E3* n = 146, *E4* carriers n = 48; BMI ≥ 25.0 kg/m^2^; *E2* carriers n = 13, *E3/E3* n = 81, *E4* carriers n = 33; BP BMI ≤ 24.9 kg/m^2^; *E2* carriers n = 33, *E3/E3* n = 145, *E4* carriers n = 48; BMI ≥ 25.0 kg/m^2^; *E2* carriers n = 13, *E3/E3* n = 80, *E4* carriers n = 33; NEFA BMI ≤ 24.9 kg/m^2^; *E2* carriers n = 33, *E3/E3* n = 145, *E4* carriers n = 48; BMI ≥ 25.0 kg/m^2^; *E2* carriers n = 12, *E3/E3* n = 80, *E4* carriers n = 32; CRP BMI ≤ 24.9 kg/m^2^; *E2* carriers n = 33, *E3/E3* n = 147, *E4* carriers n = 48; BMI ≥ 25.0 kg/m^2^; *E2* carriers n = 13, *E3/E3* n = 80, *E4* carriers n = 33; Adiponectin and uric acid BMI ≤ 24.9 kg/m^2^; *E2* carriers n = 30, *E3/E3* n = 135, *E4* carriers n = 45; BMI ≥ 25.0 kg/m^2^; *E2* carriers n = 12, *E3/E3* n = 66, *E4* carriers n = 30. Abbreviations: A/G fat % ratio—android to gynoid fat % ratio, BMI—body mass index, CRP—C-reactive protein, HC—hip circumference, HDL-C—high-density lipoprotein cholesterol, LDL-C—low-density lipoprotein cholesterol, MUFA—monounsaturated fatty acids, NEFA—non-esterified fatty acids, PUFA—polyunsaturated fatty acids, SFA—saturated fatty acids, TC—total cholesterol, TAG—triacylglycerol, VAT—visceral adipose tissue, WC—waist circumference.

**Table 3:** Participant habitual dietary intake according to *APOE* genotype in normal and overweight/obese BMI subgroups.

	BMI < 24.9 kg/m^2^ (n = 225)				BMI ≥ 25.0 kg/m^2^ (n = 125)			
	*E2* Carriers (n = 33)	*E3/E3* (n = 140)	*E4* Carriers (n = 45)	*p*	*E2* Carriers (n = 11)	*E3/E3* (n = 79)	*E4* Carriers (n = 32)	*p*
Dietary intake								
Energy intake (MJ)	7.8 ± 0.4 ^a^	8.3 ± 0.2 ^ab^	9.0 ± 0.3 ^b^	0.04	7.8 ± 0.7	9.0 ± 0.3	8.4 ± 0.4	0.17
Total fat (% TE)	38.7 ± 1.5	35.9 ± 0.7	36.1 ± 1.3	0.24	34.3 ± 2.7	36.9 ± 1.0	37.4 ± 1.6	0.60
SFA (%TE)	13.7 ± 0.7	12.8 ± 0.4	12.3 ± 0.6	0.32	12.2 ± 1.4	13.5 ± 0.6	13.0 ± 0.9	0.70
MUFA (% TE)	14.5 ± 0.7	13.5 ± 0.3	13.8 ± 0.6	0.44	12.3 ± 1.1	13.8 ± 0.4	13.9 ± 0.7	0.44
PUFA (%TE)	6.5 ± 0.4	6.2 ± 0.2	6.9 ± 0.3	0.17	5.7 ± 0.6	5.9 ± 0.2	6.5 ± 0.4	0.35
Trans fat (% TE)	0.62 ± 0.05 ^b^	0.52 ± 0.02 ^ab^	0.47 ± 0.04 ^a^	0.05	0.48 ± 0.10	0.57 ± 0.04	0.61 ± 0.06	0.50
Total CHO (% TE)	41.3 ± 1.8 ^b^	47.5 ± 0.9 ^a^	46.9 ± 1.6 ^ab^	0.01	45.3 ± 3.5	44.6 ± 1.3	42.1 ± 2.0	0.53
Total sugars (% TE)	17.7 ± 1.1	19.1 ± 0.5	19.7 ± 1.0	0.33	16.8 ± 2.2	17.7 ± 0.8	18.2 ± 1.3	0.86
Total fibre (AOAC, g)	22.6 ± 1.6 ^b^	24.9 ± 0.8 ^ab^	28.3 ± 1.4 ^a^	0.02	20.9 ± 2.4	23.4 ± 1.0	24.4 ± 1.5	0.49
Total protein (%TE)	20.9 ± 0.9 ^b^	17.5 ± 0.5 ^a^	17.4 ± 0.8 ^a^	0.01	21.1 ± 1.7	18.6 ± 0.6	20.4 ± 1.0	0.15

Data was presented as estimated marginal means ± SE, *p* < 0.05 is considered significant *E2* carriers = *E2/E3*, *E4* carriers = *E3/E4* and *E4/E4*. Data analysed by univariate general linear model (ANCOVA) adjusted for age and sex. ^ab^ significant differences (*p* < 0.05) shown as different superscript letters. Abbreviations: AOAC—Association of official analytical chemists, CHO—carbohydrate, SFA—saturated fatty acids, MUFA—monounsaturated fatty acids, PUFA—polyunsaturated fatty acids.

## Data Availability

The data presented in this study are available on request from the corresponding author.

## Supplementary Materials

The following supporting information can be downloaded at: https://www.mdpi.com/article/10.3390/ijms23179766/s1.
