# Supplementary material for: Association between APOE Genotype with Body Composition and Cardiovascular Disease Risk Markers Is Modulated by BMI in Healthy Adults: Findings from the BODYCON Study
Source: Int J Mol Sci. 2022 Aug 29;23(17):9766. doi: 10.3390/ijms23179766 (PMC9456146; doi:10.3390/ijms23179766)
Supplement: Supplementary file 1 [file ijms-23-09766-s001.zip › ijms-1865259-supplementary.pdf]

Supplementary Table S1. Characteristics of the female participants including anthropometric measures, CVD risk markers and habitual dietary intakes according to *APOE* genotype[1]

	APOE genotype groups				*P*		
	**All** (n=190)	***E2*** **carriers** (n=28)	***E3/E3*** (n=121)	***E4*** **carriers** (n=38)	Genotype[2]	BMI[2]	Genotype x BMI[3]
Frequency (%)	-	14.7%	63.7%	20%			
Age (y)	42±1	45±3	41±2	40±3	0.41		
BMI (kg/m^2)	23.5±0.3	22.5±0.7	23.6±0.4	23.8±0.6	0.31		
**Anthropometric and body composition measurements**							
WC (cm)	79.2±0.8	77.9±2.0	79.6±1.0	79.2±1.7	0.76	<0.01	0.09
HC (cm)	101±1	98.0±1.8	100±1	102±2	0.15	<0.01	0.38
Body fat (%)	32.2±0.5	30.9±1.4	32.6±0.7	32.2±1.2	0.53	<0.01	0.62
Fat mass (kg)	21.1±0.6	18.9±1.5	21.5±0.7	21.8±1.3	0.27	<0.01	0.39
Lean mass (kg)	40.6±0.4	39.5±1.1	40.6±0.5	41.7±0.9	0.30	0.27	0.75
Abdominal VAT (g)	339±25	237±61	375±29	309±52	0.10	<0.01	0.44
Android fat mass (kg)	1.47±0.07	1.21±0.19	1.55±0.09	1.46±0.16	0.27	<0.01	<0.01
Android lean mass (kg)	2.80±0.03	2.72±0.09	2.79±0.04	2.90±0.07	0.26	0.48	0.76
Android fat (%)	31.7±0.9	29.8±2.2	32.3±1.0	31.5±1.9	0.56	<0.01	0.76
Gynoid fat (%)	38.5±0.5	37.1±1.3	38.9±0.6	38.8±1.2	0.49	<0.01	0.59
A/G fat % ratio	0.80±0.01	0.79±0.04	0.81±0.02	0.78±0.03	0.77	0.02	0.66
**Cardiovascular disease risk markers**							
Blood pressure (mmHg)							
Systolic	117±1	113±3	118±1	118±2	0.27	0.15	0.01
Diastolic	70±1	68±2	71±1	71±2	0.24	0.56	0.83
Pulse pressure	47±1	46±2	47±1	47±2	0.73	0.22	0.01
TC (mmol/l)	5.19±0.07	4.86±0.16	5.28±0.08	5.18±0.14	0.06	0.23	0.87
TAG (mmol/l)	0.90±0.03	0.84±0.08	0.91±0.04	0.90±0.07	0.76	<0.01	0.26
HDL-C (mmol/l)	1.78±0.03	1.91±0.06	1.78±0.03	1.69±0.05	0.04	0.80	0.99
LDL-C (mmol/l)	2.99±0.06	2.95±0.15[b]	3.50±0.07[a]	3.49±0.13[a]	<0.01	0.06	0.49
Non-HDL-C (mmol/l)	3.42±0.07	3.09±0.13[b]	3.56±0.06[a]	3.64±0.10[a]	<0.01	0.04	0.70
TC: HDL-C ratio	2.98±0.05	2.59±0.12	3.04±0.06	3.10±0.10	<0.01	0.06	0.89
LDL-C:HDL-C ratio	1.73±0.04	1.38±0.10[b]	1.78±0.05[a]	1.85±0.09[a]	<0.01	0.15	0.82
NEFA(μmol/l)	462±13	462±35	479±17	404±30	0.09	0.86	0.87
Glucose (mmol/l)	4.93±0.03	4.97±0.08	4.94±0.04	4.92±0.07	0.88	0.68	0.67
CRP (mg/l)	1.46±0.20	1.02±0.54	1.58±0.26	1.49±0.46	0.65	1.00	1.00
Adiponectin(μg/ml)	7.80±0.42	5.90±1.05	8.04±0.51	7.98±0.90	0.18	0.34	0.29
Uric acid(μmol/l)	242±4	249±10	243±5	241±8	0.80	0.74	0.85
**Dietary intake**							
Energy intake (MJ)	7.5±0.1	6.9±0.4[a]	7.5±0.2[ab]	8.1±0.3[b]	0.05	0.10	0.30
Total fat (%TE)	36.7±0.6	37.9±1.5	36.0±0.7	37.3±1.3	0.43	0.80	0.74
SFA (%TE)	12.9±0.3	12.7±0.7	12.8±0.4	13.0±0.6	0.95	0.63	0.64
MUFA (%TE)	13.9±0.3	14.5±0.7	13.6±0.4	14.2±0.6	0.44	0.45	0.39
PUFA (%TE)	6.3±0.1	6.8±0.4	6.1±0.2	6.7±0.3	0.09	0.73	0.23
Trans fat (%TE)	0.52±0.02	0.54±0.05	0.51±0.03	0.53±0.05	0.83	0.65	0.83
Total CHO (%TE)	45.6±0.7	41.3±1.9[a]	47.2±0.9[b]	44.4±1.6[ab]	0.02	0.68	0.74
Total sugars (%TE)	19.4±0.5	18.0±1.2	19.4±0.6	20.3±1.0	0.33	0.71	0.64

Total fibre (AOAC, g)	23.7±0.7	22.1±1.7	23.4±0.8	26.0±1.5	0.18	0.85	0.60
Total protein (%TE)	18.5±0.4	21.9±1.0[b]	17.9±0.5[a]	18.1±0.9[a]	<0.01	<0.01	<0.01

[1]Data was presented as estimated marginal means ± SE, p<0.05 is considered significant. E2 carriers= E2/E3, E4 carriers= E3/E4 and E4/E4.

[2]Data was analysed by univariate general linear model (ANCOVA) adjusted for age and sex.

[3]APOE genotype x BMI interaction by ANCOVA, adjusted for age and sex. Carrier code and BMI as fixed factors and variable of interest as dependent variable.

abc significant differences (P<0.05) shown as different superscript letters

Sample sizes are as follows: for NEFA all n=189 *APOE2* carriers n=28, *APOE3/E3* n=120, *APOE4* carriers n=38; for CRP all n=189, *APOE2* carriers n=28, *APOE3/E3* n=120, *APOE4* carriers n=38; for Adiponectin and Uric acid all n=189 *APOE2* carriers n=28, *APOE3/E3* n=120, *APOE4* carriers n=38, Dietary Intakes all n=185 *APOE2* carriers n=28, *APOE3/E3* n=117, *APOE4* carriers n=37.

Abbreviations: AOAC: Association of official analytical chemists, A/G fat % ratio android to gynoid fat % ratio, BMI body mass index, CHO carbohydrate, CRP C-reactive protein, HC hip circumference, HDL-C high-density lipoprotein cholesterol, LDL-C low-density lipoprotein cholesterol, MUFA monounsaturated fatty acids, NEFA non-esterified fatty acids, PUFA polyunsaturated fatty acids, SFA saturated fatty acids, TC total cholesterol, TAG triacylglycerol, VAT visceral adipose tissue, WC waist circumference.

Supplementary Table S2. Characteristics of the male participants including anthropometric measures, CVD risk markers and habitual dietary intakes according to *APOE* genotype[1]

	APOE genotype				*P*		
	**All** (n=170)	***E2* carriers** (n=18)	***E3/E3*** (n=107)	***E4* carriers** (n=43)	Genotype[2]	BMI[2]	Genotype x BMI[3]
Frequency (%)	-	10.6%	62.9%	25.3%			
Age (y)	43±1	44±3[ab]	41±1[a]	48±2[b]	0.04		
BMI (kg/m²)	24.9±0.2	25.2±0.8	25.0±0.3	24.7±0.5	0.88		
**Anthropometric and body composition measurements**							
WC (cm)	90.1±0.8	90.7±2.2	90.2±0.9	89.3±1.4	0.82	<0.01	0.20
HC (cm)	103±1	104±2	103±1	102±1	0.70	<0.01	0.97
Body fat (%)	23.8±0.6	25.5±1.6	23.8±0.7	23.0±1.1	0.42	<0.01	0.71
Fat mass (kg)	19.5±0.6	21.1±1.8	19.5±0.7	18.8±1.2	0.56	<0.01	0.82
Lean mass (kg)	57.3±0.6	57.7±1.7	57.0±0.7	58.1±1.1	0.66	0.14	0.30
Abdominal VAT (g)	889±51	977±137	912±56	808±90	0.50	<0.01	0.60
Android fat mass (kg)	1.76±0.08	1.96±0.23	1.78±0.09	1.65±0.15	0.49	<0.01	0.79
Android lean mass (kg)	3.90±0.04	3.89±0.12	3.86±0.05	4.03±0.08	0.20	0.16	0.33
Android fat (%)	29.2±0.9	32.0±2.7	30.0±1.1	27.2±1.8	0.29	<0.01	0.69
Gynoid fat (%)	24.9±0.5	26.5±1.6	24.8±0.7	24.6±1.1	0.60	0.02	0.62
A/G fat % ratio	1.14±0.02	1.20±0.06	0.15±0.02	1.07±0.04	0.10	0.06	0.82
**Cardiovascular disease risk markers**							
Blood pressure (mmHg)							
Systolic	124±1	124±3	124±2	123±2	0.71	0.02	<0.01
Diastolic	74±1	72±2[ab]	75±1[a]	71±1[b]	<0.01	0.36	0.50
Pulse pressure	50±1	52±2	49±1	52±2	0.96	0.06	0.11
TC (mmol/l)	5.12±0.09	4.64±0.24	5.14±0.10	5.28±0.16	0.08	0.65	0.94
TAG (mmol/l)	1.07±0.04	1.25±0.12	1.01±0.05	1.11±0.08	0.13	0.02	0.01
HDL-C (mmol/l)	1.51±0.03	1.38±0.09	1.53±0.04	1.50±0.06	0.27	0.47	0.27
LDL-C (mmol/l)	3.13±0.08	2.70±0.21	3.16±0.09	3.28±0.14	0.07	0.74	0.99
Non-HDL-C (mmol/l)	3.62±0.08	3.27±0.22	3.62±0.09	3.78±0.15	0.17	0.70	0.97
TC: HDL-C ratio	3.54±0.08	3.55±0.24	3.52±0.10	3.63±0.16	0.84	0.70	0.97
LDL-C:HDL-C ratio	2.18±0.07	2.07±0.21	2.19±0.09	2.27±0.14	0.71	0.91	0.99
NEFA(μmol/l)	326±19	337±57[ab]	285±23[a]	424±37[b]	<0.01	0.02	0.15
Glucose (mmol/l)	5.15±0.04	5.01±0.12	5.16±0.05	5.15±0.08	0.50	0.02	0.19
CRP (mg/l)	1.07±0.11	1.07±0.37	1.38±0.15	0.95±0.24	0.29	0.17	0.94
Adiponectin(μg/ml)	4.77±0.34	4.69±1.04	4.70±0.44	4.85±0.65	0.98	0.98	0.78
Uric acid(μmol/l)	322±5	339±15	320±6	321±9	0.47	0.87	0.52
**Dietary intake**							
Energy intake (MJ)	9.6±0.2	8.8±0.6	9.8±0.2	9.5±0.4	0.30	0.58	0.80
Total fat (%TE)	36.5±0.7	36.8±2.4	36.6±1.0	36.0±1.5	0.94	0.18	0.86
SFA (%TE)	13.1±0.4	14.3±1.3	13.3±0.5	12.2±0.8	0.33	0.67	0.93
MUFA (%TE)	13.6±0.3	13.1±1.0	13.6±0.4	13.5±0.7	0.90	0.06	0.15
PUFA (%TE)	6.2±0.2	5.4±0.6	6.1±0.2	6.9±0.4	0.07	0.31	0.43
Trans fat (%TE)	0.57±0.03	0.65±0.08	0.57±0.03	0.54±0.05	0.48	0.14	0.16
Total CHO (%TE)	45.4±1.0	44.7±3.0	45.7±1.2	45.1±1.9	0.93	0.42	0.90
Total sugars (%TE)	17.6±0.6	17.1±1.8	17.8±0.7	17.6±1.1	0.94	0.17	0.02
Total fibre (AOAC, g)	25.7±0.7	22.2±2.2	25.5±0.9	27.4±1.4	0.14	0.63	0.73

Total protein (%TE)	18.3±0.4	19.2±1.3	17.8±0.5	19.3±0.8	0.25	0.86	0.75

[1]Data was presented as estimated marginal means ± SE, $p<0.05$ is considered significant. E2 carriers= E2/E3, E4 carriers= E3/E4 and E4/E4.
[2]Data was analysed by univariate general linear model (ANCOVA) adjusted for age and sex.
[3]APOE genotype x BMI interaction by ANCOVA, adjusted for age and sex. Carrier code and BMI as fixed factors and variable of interest as dependent variable.
abc significant differences ($P<0.05$) shown as different superscript letters

Sample sizes are as follows: for WC, HC, all n=169, *APOE2* carriers n=18, *APOE3/E3* n=106, *APOE4* carriers n=43; for Blood Pressure, all n=167, *APOE2* carriers n=18, *APOE3/E3* n=104, *APOE4* carriers n=43; for NEFA all n=166 *APOE2* carriers n=17, *APOE3/E3* n=105, *APOE4* carriers n=42; for Adiponectin and Uric acid all n=133 *APOE2* carriers n=14, *APOE3/E3* n=81, *APOE4* carriers n=37, Dietary Intakes all n=160 *APOE2* carriers n=16, *APOE3/E3* n=102, *APOE4* carriers n=40.

Abbreviations: AOAC: Association of official analytical chemists, A/G fat % ratio android to gynoid fat % ratio, BMI body mass index, CHO carbohydrate, CRP C-reactive protein, HC hip circumference, HDL-C high-density lipoprotein cholesterol, LDL-C low-density lipoprotein cholesterol, MUFA monounsaturated fatty acids, NEFA non-esterified fatty acids, PUFA polyunsaturated fatty acids, SFA saturated fatty acids, TC total cholesterol, TAG triacylglycerol, VAT visceral adipose tissue, WC waist circumference.

Supplemental Table S3. Participant physical activity levels according to *APOE* genotype groups[1]

	APOE genotype				*P*	
	**All** (n=301)	***E2* carriers** (n=40)	***E3/E3*** (n=186)	***E4* carriers** (n=71)	Genotype[2]	Genotype x BMI[3]
Steps/ day	9672±216	9500±594	9850±275	9287±446	0.54	0.31
Energy expended (kcal/day)	308±13	320±35	313±16	296±26	0.82	0.57
Percentage time per day spent:						
Sedentary	69.7±0.4	71.3±1.1	69.5±0.5	69.4±0.8	0.32	0.67
Performing light PA	25.5±0.4	23.7±1.0	25.6±0.5	26.1±0.8	0.13	0.75
Performing moderate to vigorous PA	4.8±0.2	5.1±0.4	4.8±0.2	4.6±0.3	0.67	0.23

[1]Data was presented as estimated marginal means ± SE, *E2* carriers= *E2/E3*, *E4* carriers= *E3/E4* and *E4/E4*.
[2]Data was analysed by univariate general linear model (ANCOVA) adjusted for age and sex.
[3]APOE genotype x BMI interaction by ANCOVA, adjusted for age and sex

Supplemental Table S4. Participant physical activity measures according to *APOE* genotype in normal and overweight/obese BMI groups[1]

	BMI <24.9 kg/m² (n = 203)				BMI ≥ 25.0 kg/m² (n = 98)			
	***E2* carriers** (n=29)	***E3/E3*** (n =127)	***E4* carriers** (n = 43)	*P*	***E2* carriers** (n=11)	***E3/E3*** (n =59)	***E4* carriers** (n = 28)	*P*[2]
Steps/ day	9527±724	10362±345	10001±595	0.56	9385±1007	8779±432	8142±629	0.53
Energy expended (kcal/day)	290±38	296±18	292±32	0.99	401±77	348±33	306±48	0.56
Percentage time per day spent:								
Sedentary	71.5±1.3	69.9±0.6	69.8±1.1	0.21	70.7±2.1	70.8±0.9	69.3±1.3	0.61
Performing light PA	23.4±1.2	25.9±0.6	25.5±1.0	0.17	24.3±1.8	25.1±0.8	26.8±1.1	0.38
Performing moderate to vigorous PA	5.06±0.52	5.14±0.25	4.92±0.43	0.91	4.96±0.83	4.14±0.36	3.97±0.52	0.60

[1]Data was presented as estimated marginal means ± SE, p<0.05 is considered significant *E2* carriers= *E2/E3*, *E4* carriers= *E3/E4* and *E4/E4*.
[2]Data analysed by univariate general linear model (ANCOVA) adjusted for age and sex.
